# A continuous pursuit dataset for online deep learning-based EEG brain-computer interface

**DOI:** 10.1038/s41597-024-04090-6

**Published:** 2024-11-20

**Authors:** Dylan Forenzo, Hao Zhu, Bin He

**Affiliations:** Department of Biomedical Engineering, Carnegie Mellon University, Pittsburgh, USA

**Keywords:** Brain-machine interface, Biomedical engineering

## Abstract

This dataset is from an EEG brain-computer interface (BCI) study investigating the use of deep learning (DL) for online continuous pursuit (CP) BCI. In this task, subjects use Motor Imagery (MI) to control a cursor to follow a randomly moving target, instead of a single stationary target used in other traditional BCI tasks. DL methods have recently achieved promising performance in traditional BCI tasks, but most studies investigate offline data analysis using DL algorithms. This dataset consists of ~168 hours of EEG recordings from complex CP BCI experiments, collected from 28 unique human subjects over multiple sessions each, with an online DL-based decoder. The large amount of subject specific data from multiple sessions may be useful for developing new BCI decoders, especially DL methods that require large amounts of training data. By providing this dataset to the public, we hope to help facilitate the development of new or improved BCI decoding algorithms for the complex CP paradigm for continuous object control, bringing EEG-based BCIs closer to real-world applications.

## Background & Summary

Brain-computer interfaces (BCIs) are devices that allow their users to communicate directly with computers or other machines using their brain activity. These systems provide an opportunity to replace or restore some motor functions for motor-impaired populations, or even improve the lives of healthy individuals by providing a direct line of communication with electronic devices and robots^1,2^.

Electroencephalography, or EEG, is a prominent method for noninvasively recording neural signals for use with BCI systems since it is portable, relatively inexpensive, and can record across the entire scalp with high temporal resolution. One of the most popular control paradigms used for EEG-based BCIs is called Motor Imagery (MI). In this setting, the BCI user will imagine the feeling or sensation of moving parts of their body without actually executing any movement, causing a predictable change in their brain activity depending on the body part that is imagined^3,4^. There are oscillations around the 8–13 Hz range in the sensorimotor area of the human brain, which is typically referred to as the alpha band in the context of EEG activity but also called the *mu* rhythm when specifically referring to the sensorimotor area. When BCI users perform MI, the magnitude of these natural oscillations in the alpha (or *mu*) band decreases/increases in a phenomenon known as Event-Related Desynchronization/Synchronization (ERD/ERS)^5^. This ERD/ERS signal can be used to decode human intention to map from imagining movement of certain body parts into control commands^6,7^.

EEG BCIs based on the MI paradigm have been successfully used to control a variety of devices, including virtual cursors^8–10^, robotic limbs^11–16^, wheelchairs^17,18^, a virtual drone^19,20^ and even a quadcopter drone^21^. These devices also have potential clinical applications for helping paralyzed and other motor impaired patients such as those with spinal cord injuries and stroke survivors. Studies have investigated using EEG-BCIs to allow tetraplegic patients to control a computer cursor^22^, virtual racing games^23,24^, and even physical devices like robotic arms and wheelchairs^17,25^. BCIs may also benefit stroke survivors with motor impairments by aiding in post-stroke rehabilitation^13,26–31^. In each of these applications, one of the main benefits of using EEG-BCIs is the non-invasive nature of EEG signal acquisition which does not require any invasive medical procedures.

A major drawback of EEG, however, is that the recorded signals have low signal-to-noise ratios and spatial resolution due to the smearing effect of the head volume conduction^32,33^. These lower quality signals can limit the performance of EEG BCI devices. To overcome these challenges, signal-processing methods are developed to extract meaningful features from EEG recordings and decode the BCI users’ intentions. Over the past several years, deep learning (DL) methods have seen a rise in popularity for signal processing in EEG BCI systems^34–38^. These DL-based decoders can achieve state-of-the-art performance on BCI tasks and are able to automatically optimize for different subjects or tasks without needing operator inputs^34,39,40^.

One of the main challenges when using DL-based methods is the requirement for large amounts of training data needed for supervised learning to achieve good decoding performance. Several recent BCI studies have helped to overcome this challenge by releasing large publicly available EEG BCI datasets that can be used to train new DL models^41,42^. Table 1 provides a summary of a few selected publicly available EEG BCI datasets and illustrates the main differences with the dataset presented here. Information on additional EEG datasets can be found in a recent review paper on DL for EEG-BCIs, including datasets on both BCI and other EEG applications^43^.

**Table 1:** An overview of some selected example EEG BCI datasets and how they differ from the data presented in this data descriptor.

Dataset Name	BCI Task	Number of Subjects	Number of Sessions	Notes	Link
BCI Competition IV Dataset 2a	4 class MI	9	2	No feedback was provided to subjects. This dataset is currently the most commonly used in the EEG BCI community and provides a benchmark for training and testing BCI decoders.	https://www.bbci.de/competition/iv/#dataset2a
Physionet EEG Motor Movement/Imagery Dataset	4 class ME/MI	109	1	Subjects performed both motor execution and motor imagery in different trials. This dataset contains a very large number of subjects, but only a single session of data with a single type of ME/MI in each trial	https://www.physionet.org/content/eegmmidb/1.0.0/
Continuous sensorimotor rhythm based brain computer interface learning in a large population	2 or 4 class MI	62	7 to 11	This is a very large BCI dataset in terms of both subjects and sessions. Subjects performed either 2 or 4 class MI with feedback in 7-11 sessions with a single type of MI target in each trial.	10.6084/m9.figshare.13123148
EEG-BCI Dataset for “Continuous Tracking using Deep Learning-based Decoding for Non-invasive Brain-Computer Interface”	Two-dimensional continuous pursuit	28	4 or 8	The dataset described by this article. This dataset differs from the other relevent datasets above by providing feedback in a large number of subjects over several sessions in the CP task, where there are many different MI targets in a single trial instead of just 1.	10.1184/R1/25360300

Even with these public datasets, BCI data is still limited compared to what is available for other fields where DL has seen success such as computer vision or natural language processing^44^, and training subject-specific models for new subjects requires running costly and time-consuming BCI sessions to collect subject-specific data. In addition, the BCI data available for MI tasks is largely from traditional BCI experiments where each trial has only a single type of MI being performed.

In contrast to traditional BCI experiments with only a single target in each trial, a recently proposed task called Continuous Pursuit (CP) consists of the BCI user tracking a randomly moving target by performing different types of MI to control a cursor in various directions throughout a single trial. This task is more complex than paradigms with only a single type of MI control per trial, since subjects are required to change their MI dynamically throughout a trial to follow the target. DL-based decoding methods could be used to potentially improve performance in this more difficult task, but the BCI data available for training is largely from traditional BCI tasks, which may not be optimal for training models for the CP task.

To investigate the effectiveness of using DL-based decoding for online CP BCI experiments, we conducted a research study of 28 human subjects performing multiple sessions of BCI using both traditional and DL-based BCI decoders. Here, we provide the large EEG BCI dataset that resulted from these CP experiments to the public, for training and developing BCI decoders for the CP task. This dataset consists of approximately 168 hours of EEG recordings from CP experiments from 28 different human subjects that performed either 4 or 8 sessions of BCI experiments, depending on the sub-study^45^. This dataset may be particularly useful for training both subject-specific DL models, or inter-subject generalized models due to the large amount of both subject-specific data (up to 8 sessions or 8 hours of EEG data per subject) and the number of unique subjects (n = 28). By providing this dataset, we hope to enable interested groups to develop new or improved BCI decoding methods for the CP task. Improving performance in this task can lead to increased continuous control of BCI devices like robotic limbs and can bring these devices closer to real-world or clinical applications.

The original publication for this research study presents the results and outcome from the experiments, as well as providing an in-depth description of the experiment protocol and methods. The purpose of this Scientific Data article is focused on the description of the dataset that resulted from those experiments and provides several new analyses of the dataset to show its value for training BCI decoders. This publication includes specific descriptions of all the dataset fields, a comparison between BCI decoders used in the original study vs. chance level, a comparison of grouped DL-based models against the traditional method, and an analysis of using DL with and without electrodes that are susceptible to eye artifacts, all of which are not present in the original publication describing the study and are unique to this article.

## Methods

### Subject recruitment

A total of 30 healthy human subjects were recruited for this study, including 15 for each of the two sub-studies. Fourteen subjects completed the entire study protocol in each sub-study, resulting in the 28 subjects included in this dataset. The subjects were unique for each sub-study and no subject participated in both sets of experiments. All experiments were approved by the institutional review board at Carnegie Mellon University (under protocol STUDY2017_00000548) and informed consent for the experimental procedure, including data sharing, was obtained from each subject via written consent form. The average age for all subjects recruited was 23.67 years old, with 27 subjects being right-handed and 16 males. For the first sub-study, the average age was 25.2 years old with 14 right-handed and 8 males. In the second sub-study, the average age was 22.23 years old with 13 right-handed and 8 males.

### Experimental procedure

This dataset was originally produced from a study investigating the use of DL for online CP BCIs^45^. This original study was split into two sub-studies, each with 14 subjects. In the first sub-study, human subjects were recruited for up to 8 sessions of BCI experiments using both DL-based and a traditional decoder for the CP task. After each session, the DL-based decoders were updated using all the available subject-specific data for training, so that the models were trained with increasing amounts of data as the study progressed. The goal of this investigation was to demonstrate the use of DL-based decoding in online CP experiments and to characterize how the supply of subject-specific training data affects DL performance in the CP task.

A second sub-study was performed after these initial 8 sessions, where a new cohort of subjects was studied for 4 sessions of CP BCI experiments. In this second sub-study, a generalized DL model, referred to as the “base model”, was trained using the data from all the subjects from the first sub-study to produce a DL model for CP BCI that was generalized across subjects. This “base model” was then fine-tuned for each new subject using subject-specific data after each session in the second sub-study. The goal of this second sub-study was to investigate if transfer learning (TL) techniques such as fine-tuning (referred to as recalibration in the original manuscript) could improve performance in early sessions for new BCI users.

In each BCI experiment, subjects performed Motor Imagery (MI) to control the BCI device. In this paradigm, the subjects were asked to imagine the feeling or sensation of moving either their left hand to move the cursor to the left, their right hand to move the cursor to the right, both hands simultaneously to move the cursor upwards, and no hands (or rest) to move the cursor downwards. The subjects were also told that they could move the cursor in any direction in the 2D workspace by combining these four cardinal movements. It was up to each subject to determine the best way for them to combine the MI strategies to generate their best control. In the CP task, a small circular target was made to randomly drift around the square 2D workspace. The subjects’ goal during each trial was to use the MI paradigm to move another small virtual circle, called the cursor, to stay as close as possible to the virtual target. Each trial consisted of 60 seconds of tracking the virtual target, with a small inter-trial break for a few seconds, before the next trial began with both the cursor and target reset to the center of the workspace. Trials were performed in blocks of 5 trials called runs (about 5 minutes of BCI each), and 12 runs of BCI were performed each session.

### Data acquisition

Subjects were seated in a comfortable chair in front of a large television monitor. The exact distance to the screen was not measured or enforced, but all subjects were seated in the same general area relative to the screen. Brain signals were recorded using 64-channel Neuroscan Quik-caps with the SynAmps/RT amplifiers (Compumedics Neuroscan, NC). The caps were centred on subjects’ heads by placing the central Cz electrode midway between the nasion and inion and midway between the tips of the ears using a measuring tape. Electrode impedances were reduced below 5 kΩ at the start of each session using EEG compatible electrode gel. EEG signals were recorded at 1000 Hz and filtered between 0.1 and 200 Hz. An additional notch filter was applied at 60 Hz to remove artifacts from nearby AC devices. The EEG signals were sent to the online signal processing software in blocks every 40 ms for analysis and storage.

### BCI data

The filtered EEG signals were sent from the Neuroscan recording software to BCI2000^46^ over a socket for signal processing and analysis in blocks every 40 ms. Several different signal processing methods were used in this study, referred to as decoders, which fall into two categories: either the traditional method, or DL-based methods. Each run of CP BCI performed in this study used a single decoder throughout the entire run. The decoder used for each run had a direct effect on the online BCI performance for that run, and so the decoder used for each run is labelled in the dataset presented here.

### Traditional decoder

The traditional decoder uses the ARSignalProcessing module included in BCI2000 and has been used extensively in BCI research. This decoder uses an autoregressive model of the data to estimate the spectral power in the high alpha band (10.5-13.5 Hz) of the signal in specific electrodes. The high alpha band power in the C3 and C4 electrodes is then used in a linear classifier to produce a cursor velocity in the horizontal (C4-C3) or vertical (-C3-C4) directions. More information on this algorithm is available either in the BCI2000 online documentation or described in previous BCI works^11,41^.

### DL-based decoders

The DL-based methods were implemented in BCI2000 using the BCPy2000 modules with custom Python scripts. The implementation of these decoders is summarized here briefly, but detailed descriptions of each of the DL-based decoders can be found in the paper describing the original study. First, DL models are trained offline using BCI data recorded from previous CP sessions. The various DL methods differed in either the architecture (EEGNet vs PointNet) or the amount and type of data (subject-specific or inter-subject) used to train the models (DL vs TL vs recalibration). The models were trained using supervised learning, with the vector between the recorded cursor and target positions used as the label for training. An example of how to calculate this label for the provided dataset is given in the Usage Notes section below. The learned model weights were then used for the next online experimental session. During an online CP BCI run with a DL-based decoder, the EEG signal was downsampled by a factor of four and then pre-processed using a Common Average Reference (CAR) filter and exponential moving standardization. The pre-processed data window was then run through the DL model to produce two numbers: a horizontal and a vertical velocity used to control the cursor.

## Data Records

### Data access

This dataset was uploaded to the Figshare (https://figshare.com) platform and can be accessed at: 10.1184/R1/25360300)^47^.

### Data structure

The original study producing this dataset consists of two distinct sub-studies, as described in the Methods section. Details on these two sub-studies, including subject information and which decoders were used can be found in Table 2.

**Table 2:** Details for each of the sub-studies in the original study that produced this dataset.

Sub-study	Number of Subjects	Number of Sessions	Decoders
Main	14	8	Traditional (AR), EEGNet (EG), PointNet (PN)
Transfer Learning	14	4	Traditional (AR), EEGNet (DL), EEGNet with TL (TL), EEGNet with recalibration (CL)

The data presented here consists of MATLAB files (‘.mat’) where each individual file includes the EEG data and other information for a single run of CP BCI. The runs are grouped by subjects and collected into ‘.zip’ files for convenience. In addition to the EEG time series, the MATLAB files contain other information, including descriptive information like the subject, session, run number, as well as other BCI information like the cursor and target positions throughout the trials. A full list of all the sub-fields included in the MATLAB structs is shown in Table 3, along with the data type and a brief description of each of the fields. A similar list is also provided in the README file included with the dataset in the Figshare repository, which also provides more detailed descriptions of each of the struct fields.

**Table 3:** Descriptions of each field of the MATLAB structs included in the dataset.

Field	Data Type	Brief Description
data	Matrix of double	A matrix containing the EEG values for the run.
times	Vector of double	A vector containing the timestamps corresponding to the EEG data points in “data”.
event	Struct	A structure containing the event information for the run. There are two types of events: TrialStart and TrialEnd, corresponding to the start and end of each trial.
subject	String	The subject ID in the format “S##” (ex. “S01”, “S13”).
session	String	The session ID in the format “Se##” (ex. “S04”).
run	String	The run ID in the format “R##” (ex. “R02”).
decoder	String	The decoder ID as a two-letter abbreviation (ex. “EG”). See Table 4 for full decoder names.
fs	Double	Sampling frequency, 1000 Hz for all runs.
study	String	A string identifying which sub-study the data is from. Either “Main” or “Transfer Learning”.
cursorpos	Struct	A structure containing the horizontal and vertical cursor positions as timeseries. Cursor and target positions are sampled (updated) at 25 Hz.
targetpos	Struct	A structure containing the horizontal and vertical target positions as timeseries. Cursor and target positions are sampled (updated) at 25 Hz.
postimes	Vector of double	A vector of timestamps corresponding to when each of the cursor/target positions were recorded. Cursor and target positions are sampled (updated) at 25 Hz.
channellabels	Vector of cells	A vector of cells where each cell contains the label for the corresponding channel in “data”.
cursorvel	Struct	A structure containing the horizontal and vertical cursor velocities as timeseries. These velocities are the scaled outputs from the online decoders.

More detailed descriptions can be found in the README file included with the dataset on Figshare.

A major component of the original study was to test a several different traditional and DL-based BCI decoders for online CP experiments. The decoder used in each online run can have a large effect on the online performance of that run, and different decoders can have different performances across subjects. The label of which decoder was used for the online experiments in each run is included under the ‘decoder’ field. These labels are given as two-letter abbreviations, and the full names and a brief description of each decoder can be found in Table 4.

**Table 4:** Abbreviations and descriptions for each of the BCI decoders used in this study.

Decoder	Abbreviation	Description
Traditional (AutoRegressive)	AR	Traditional method, autoregressive power spectrum estimation from BCI2000.
EEGNet	EG	Standard EEGNet in the “Main” sub-study.
PointNet	PN	Standard PointNet.
EEGNet (sub-study 2)	DL	Standard EEGNet in the “Transfer Learning” sub-study. Same as “EG” above.
Transfer Learning	TL	EEGNet with transfer learning.
reCalibration	CL	EEGNet with recalibration.

The decoder used for each of the online BCI runs is included in the dataset as a field in the MATLAB struct.

### Data fields

The data field contains the EEG timeseries as a matrix with size 62 (the number of channels/electrodes) by the number of time points in the run. This matrix contains data from both the active 60 second CP trials, as well as the short inter-trial segments between trials. The EEG signals were minimally pre-processed with a band pass filter between 0.1-200 Hz and a notch filter at 60 Hz. No other artifact removal or filtering was performed. The data is given close to raw form in order to give interested parties the most freedom to choose how to process the data for their specific applications.

The times field provided the time stamps corresponding to each of the EEG data points given in data in terms of milliseconds. Each of the 62 EEG channels are labelled in channellabels in the same order they appear in the data field (ex. the 6^th^ label in channellabels corresponds to the 6th row of data).

Each BCI run is made up of five (5) trials of CP BCI, with short inter-trial intervals between each trial. The event field is a structure containing information on each of the trials in the run. Each event is either a TrialStart or TrialEnd event, corresponding to the start or end of each trial. These events are also structs with three fields each: latency, which gives the timestamp of the start of the event, duration which is the length of the event in milliseconds, and type denoting either TrialStart or TrialEnd. An example of how to extract only the active portions of trials using this information is provided in the Usage Notes section below.

In addition to the EEG data, descriptive information about each run is also included in the MATLAB files. The subject, session, and run fields provide information about the subject, session, and run number for that file, respectively. Each of these labels is given in as a prefix (‘S’ for subject, ‘Se’ for session, ‘R’ for run) followed by a two-digit number. If the subject/session/run is less than 10, it is prefixed with a ‘0’. For example, subject 1 is ‘S01’, session 3 would be ‘Se03’, etc. while subject 15 is given as ‘S15’.

Other descriptive fields include fs which is the sampling frequency (always 1000 Hz for each run), and study, which denotes which sub-study the run belongs to. Runs from the 14 subjects studied in the first sub-study will be labelled as “Main” in the study field, while the 14 subjects studied in the second sub-study are labelled as “Transfer Learning”. The decoder labels are also provided as two-letter abbreviations in the decoder field, and the matching full names are displayed in Table 4. More detailed information about each of these decoders can be found in the paper describing the original study.

Lastly, information from the CP application is provided in the cursorpos, targetpos, cursorvel, and postimes fields. Each of the cursorpos, targetpos, and cursorvel fields are structs containing two fields: x and y which are the horizontal and vertical components of the 2D vectors. The cursorpos field is a 2D vector giving the horizontal and vertical cursor positions throughout the run, while targetpos contains the same information for the target. Similarly, cursorvel is a 2D vector giving the horizontal and vertical cursor velocities. These cursor velocities are the scaled output of the online decoder used to control the cursor for each run. Lastly, postimes provides the timestamps corresponding to each of the cursor and target positions, as well as the cursor velocities. While the EEG signals were sampled at 1000 Hz, the cursor and target positions / velocities were only updated at 25 Hz since data packets were processed every 40 ms during the online experiment.

## Technical Validation

In general, the performance of the decoders in the online experiments of the original study validates the reliability of the MI signals in this dataset. Each of the decoders reached a performance above chance level that was shown to be statistically significant^45^. This shows that the MI features produced by subjects in the original study are reliable and can be decoded from the EEG signals included in this dataset. This was validated in online experiments with real-time BCI control, with decoders that used only a subset of electrodes around the sensorimotor cortex (the traditional [AR] method in the original study) and using all the available electrodes (the DL models in the original study).

**Fig. 1:**
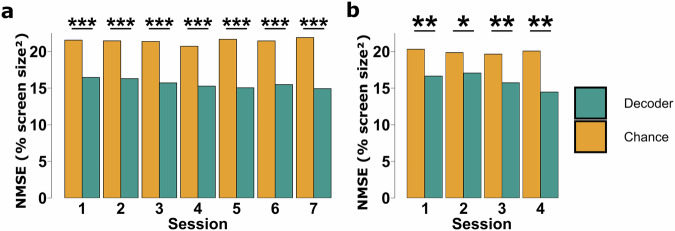
Performance of BCI decoders compared to chance level in each session. (**a**) Results from sub-study 1 with 14 subjects over 7 sessions. (**b**) Results from sub-study 2 with 14 subjects over 4 sessions. Statistics are from Wilcoxon Signed Rank tests comparing subject-averaged performance (n = 14) for both decoders in each session. P-values were adjusted using the Holm method and are displayed as: *p < 0.05, **p < 0.01, ***p < 0.001.

**Fig. 2:**
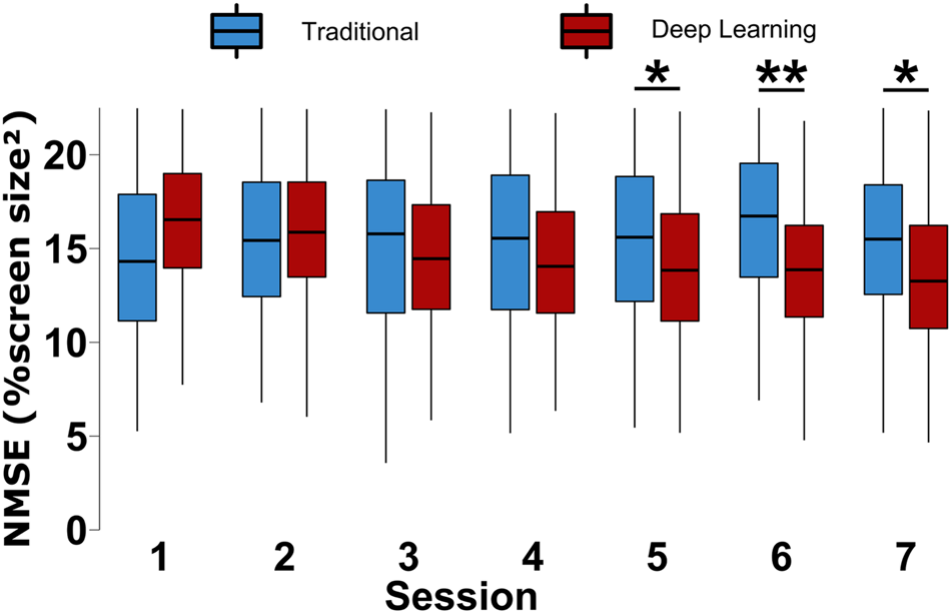
Comparison of DL-based decoders and traditional AR method throughout sub-study 1. Both the DL-based and traditional methods begin with similar performance in early sessions, but the DL-based methods begin to outperform the AR decoder as more training data becomes available. After the fourth session, there is a statistically significant difference between the performance of the two decoder groups. Statistics are from Wilcoxon Signed Rank tests with p-values adjusted using the Holm method. P-values are displayed as: *p < 0.05, **p < 0.01, ***p < 0.001. Boxplots are drawn in the style of Tukey using the median, first, and third quantiles with whiskers up to 1.5 times the inter-quartile range.

Figure 1 shows the average online performance across all the decoders in each session of both the first sub-study (Fig. 1a) and the second sub-study (Fig. 1b), compared to chance level runs. The performance metric in these plots is the Mean Squared Error (squared distance) between the cursor and target throughout a trial, normalized by the screen diagonal so that the maximum value is 1, or 100%. For this metric, a lower value corresponds to better performance as the cursor is close to the target throughout the trial. In each session, the BCI decoders perform significantly better than chance level (Wilcoxon signed rank test), showing that the MI features in this dataset are suitable for BCI decoding applications and may be useful for training future DL models. Additionally, the results in Fig. 2 show the performance of the traditional AR method compared to the average performance between both DL models (EEGNet and PointNet) in each session. While both methods achieve similar performance in early sessions, the DL models eventually outperform the traditional decoder after the 4^th^ session (Wilcoxon signed rank test). This provides an example of how DL-based decoders have the potential to improve BCI performance, and why more training data is important for developing these types of models.

Another potential concern is that subjects may introduce eye movement artifacts into the EEG recordings when they look around the workspace during the CP trials. These eye movement artifacts have the potential to artificially boost the BCI performance of DL models, if the models can associate the large changes in the EEG signals with eye movements towards the target.

**Fig. 3:**
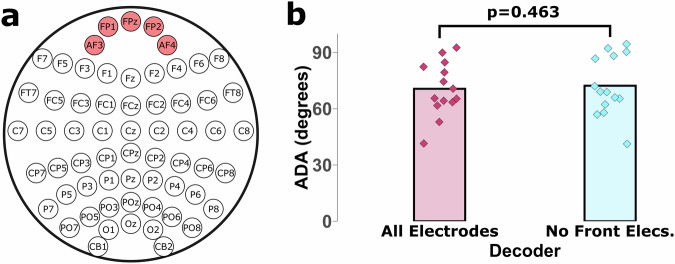
Eye artifacts in DL models. (**a**) The electrode montage used in the original study with the five frontal electrodes highlighted. The frontal electrodes are particularly susceptible to eye movement artifacts being located closer to the eyes. (**b**) To test if eye artifacts significantly impacted the results of DL performance in this dataset, an EEGNet model was trained with all electrodes and without the frontal electrodes highlighted in Panel A. No significant difference was found between the performance of the models, suggesting that any eye artifacts in the dataset do not substantially affect decoding performance. Statistical testing was performed with a Wilcoxon Signed Rank Test.

To demonstrate the robustness of the MI features compared to potential eye movement artifacts, an EEGNet model was trained and tested offline for two conditions using the data from all subjects in the first sub-study. The first condition used all 62 of the available electrodes as a control. In the second condition, the five frontal electrodes shown in Fig. 3a were removed from the dataset and the EEGNet model was trained with 57 channels as inputs. These five frontal electrodes are located closer to the eyes and are therefore particularly susceptible to eye movement artifacts. The results, shown in Fig. 3b, show that there is no substantial difference in decoding performance whether the frontal electrodes were included. This provides evidence that any potential eye movement artifacts in the data are not overshadowing the MI features produced by subjects during the experiments. This validates the robustness of this dataset as a resource for training and developing new algorithms for decoding MI features for the CP BCI task.

## Usage Notes

### Importing the data

The dataset is provided in MATLAB file format (‘.mat’). This filetype can be readily loaded into MATLAB using the “load” function. However, MATLAB software is not required to use this data, and it can be loaded into Python without any modification using the “mat73” Python package. This package can be installed using pip and can also be installed into a conda environment by first activating the environment, installing pip into the environment, then using pip in the environment to install “mat73”. To load one of the MATLAB files into Python using this package, after importing type: *data* = *mat73.loadmat(‘FileToLoad.mat’).*

### Extracting trials from the dataset

In the study design used for CP experiments, each trial lasts 60 seconds, followed by a brief inter-trial segment where the subject is asked to rest for a few seconds. The EEG data included with this dataset contains both the active 60 second portions of each trial, as well as the inter-trial intervals. In many cases, it may be desired to only use the active 60 second portions of the data. In order to extract these segments, the ‘event’ field is included, which contains the ‘TrialStart’ and ‘TrialEnd’ events. Each of these events also includes a field called ‘latency’, which is the time stamp when the event starts, and ‘duration’, which is the length of the event in milliseconds. To only extract the active feedback portions of the trials, one option would be to only take the data from the start of each ‘TrialStart’ event (the timestamp given in ‘latency’) to the end of the ‘TrialStart’ event (‘latency’ + ‘duration’).

### Labelling data for supervised learning applications

The CP task is not directly well-suited for supervised learning algorithms such as machine learning and DL techniques, due to the lack of a clear label for the data. In other BCI tasks where DL has previously been applied, the subject is often only asked to perform one type of MI in each trial (for an example, see the popular BCI Competition IV 2a dataset^42^). In contrast, subjects need to perform a variety of different MI controls in each CP trial, and often will need to dynamically switch between these controls to move in different directions. One option to overcome this challenge is to assume that the subject is performing MI to move in the ideal direction for the task. In the CP case, this translates to using the cursor-to-target vector as the label for the data.

To calculate this label, first cut the EEG data into windows of some constant size. For each data window, take the average cursor and target positions throughout the window (or for a slightly different labelling system, take the final cursor and target positions in the window instead) and calculate the vector between them by subtracting:

1 $${Label}=({T{arget}}_{x}-{C{ursor}}_{x},\,{T{arget}}_{y}-{C{ursor}}_{y})$$

This vector can then be used as a ‘label’ for supervised learning. It can be interpreted as the optimal direction (and magnitude if not normalized) for the subject to move their cursor in order to get closer to the target. If the subject is attempting the CP task correctly, they should be attempting to perform MI corresponding to this direction, or close to it.

### Example applications of the dataset

One potential application of this dataset is to be used to develop either new DL architectures specifically for the CP BCI task, or to implement or modify already existing architectures for this paradigm instead. The original study of this dataset focused on two DL architectures: the EEGNet model and a modified version of the PointNet architecture. Although EEGNet remains a popular DL architecture for BCI and other EEG applications, it is a relatively simple DL architecture that was first developed almost a decade before this study. Recent advances made in DL since that time could provide new opportunities to improve BCI performance and capabilities in the CP task. Some examples of recently developed methods that could be modified or implemented for CP BCI could include: transformers like EEGConformer^40^, GPTs such as NeuroGPT^48^, and many others^43^. The dataset presented here could be used as training data to implement these models for the CP task, and to both validate and compare the performances of various implementations. The PointNet model used in the original study is one example of how a DL architecture from another field can be implemented for the CP BCI task, but there is much more room for exploration on this topic.

When using the data to develop or implement new DL models for the CP task, implementing cross-validation of the dataset may be useful to ensure that results are robust across different testing sets. This method involves splitting the dataset into several partitions and re-training models several times using different partitions for training and testing. For the dataset presented here, cross-validation could be used with different subject groups when training subject-independent models or could be used to split a single subjects’ data into multiple partitions.

Another potential use of this dataset is to investigate the differences between continuous pursuit data and the data recorded from tasks with discrete trials. It may be interesting to explore the effects of using discrete task data to train DL models for the CP task, and also vis-a-versa to use models trained on the more complex CP task for discrete BCI tasks. The dataset introduced in this article provides a means for performing future offline analyses such as these.

## Data Availability

Code relevant to the analysis of this dataset is available at: https://github.com/bfinl/CPDL. This includes the EEGNet and PointNet architectures used in the original analysis and online experiments that produced this dataset, as well as an example DL training script and the electrode positions for the Neuroscan 64-channel Quik-Caps.

## References

[1] He, B., Yuan, J., Meng, J. & Gao, S. Brain-Computer Interfaces. in *Neural Engineering* 131–183 (Springer International Publishing, Cham, 2020).

[2] Edelman, B. J. *et al*. Non-invasive Brain-Computer Interfaces: State of the Art and Trends. *IEEE Reviews in Biomedical Engineering*10.1109/RBME.2024.3449790 (2024). 10.1109/RBME.2024.3449790 PMC11861396 39186407

[3] Pfurtscheller, G. & Neuper, C. Motor imagery and direct brain-computer communication. *Proceedings of the IEEE* **89**, 1123–1134 (2001).

[4] Yuan, H. *et al*. Negative covariation between task-related responses in alpha/beta-band activity and BOLD in human sensorimotor cortex: An EEG and fMRI study of motor imagery and movements. *NeuroImage* **49**, 2596–606 (2010). 19850134 10.1016/j.neuroimage.2009.10.028 PMC2818527

[5] Pfurtscheller, G. & Lopes da Silva, F. H. Event-related EEG/MEG synchronization and desynchronization: basic principles. *Clinical Neurophysiology* **110**, 1842–1857 (1999). 10576479 10.1016/s1388-2457(99)00141-8

[6] Yuan, H. & He, B. Brain–Computer Interfaces Using Sensorimotor Rhythms: Current State and Future Perspectives. *IEEE Transactions on Biomedical Engineering* **61**, 1425–1435 (2014). 24759276 10.1109/TBME.2014.2312397 PMC4082720

[7] He, B., Baxter, B., Edelman, B. J., Cline, C. C. & Ye, W. W. Noninvasive Brain-Computer Interfaces Based on Sensorimotor Rhythms. *Proceedings of the IEEE* **103**, 907–925 (2015). 34334804 10.1109/jproc.2015.2407272 PMC8323842

[8] Wolpaw, J. R. & McFarland, D. J. Control of a two-dimensional movement signal by a noninvasive brain-computer interface in humans. *Proceedings of the National Academy of Sciences* **101**, 17849–17854 (2004). 10.1073/pnas.0403504101 PMC535103 15585584

[9] Wolpaw, J. R., McFarland, D. J., Neat, G. W. & Forneris, C. A. An EEG-based brain-computer interface for cursor control. *Electroencephalography and Clinical Neurophysiology* **78**, 252–259 (1991). 1707798 10.1016/0013-4694(91)90040-b

[10] Stieger, J. R. *et al*. Mindfulness Improves Brain–Computer Interface Performance by Increasing Control Over Neural Activity in the Alpha Band. *Cereb Cortex* **31**, 426–438 (2020). 10.1093/cercor/bhaa234 PMC7727383 32965471

[11] Meng, J. *et al*. Noninvasive Electroencephalogram Based Control of a Robotic Arm for Reach and Grasp Tasks. *Sci Rep* **6**, 38565 (2016). 27966546 10.1038/srep38565 PMC5155290

[12] Edelman, B. J. *et al*. Noninvasive neuroimaging enhances continuous neural tracking for robotic device control. *Science Robotics* **4**, eaaw6844 (2019). 31656937 10.1126/scirobotics.aaw6844 PMC6814169

[13] Ang, K. K. *et al*. A Randomized Controlled Trial of EEG-Based Motor Imagery Brain-Computer Interface Robotic Rehabilitation for Stroke. *Clin EEG Neurosci* **46**, 310–320 (2015). 24756025 10.1177/1550059414522229

[14] Mondini, V., Kobler, R. J., Sburlea, A. I. & Müller-Putz, G. R. Continuous low-frequency EEG decoding of arm movement for closed-loop, natural control of a robotic arm. *J. Neural Eng.* **17**, 046031 (2020). 32679573 10.1088/1741-2552/aba6f7

[15] Cao, L. *et al*. A brain-actuated robotic arm system using non-invasive hybrid brain–computer interface and shared control strategy. *J. Neural Eng.* **18**, 046045 (2021). 10.1088/1741-2552/abf8cb 33862607

[16] Xu, B. *et al*. Continuous Hybrid BCI Control for Robotic Arm Using Noninvasive Electroencephalogram, Computer Vision, and Eye Tracking. *Mathematics* **10**, 618 (2022).

[17] Tonin, L. *et al*. Learning to control a BMI-driven wheelchair for people with severe tetraplegia. *iScience* **25**, 105418 (2022). 36590466 10.1016/j.isci.2022.105418 PMC9801246

[18] Galán, F. *et al*. A brain-actuated wheelchair: Asynchronous and non-invasive Brain–computer interfaces for continuous control of robots. *Clinical Neurophysiology* **119**, 2159–2169 (2008). 18621580 10.1016/j.clinph.2008.06.001

[19] Royer, A. S., Doud, A. J., Rose, M. L. & He, B. EEG Control of a Virtual Helicopter in 3-Dimensional Space Using Intelligent Control Strategies. *IEEE Transactions on Neural Systems and Rehabilitation Engineering* **18**, 581–589 (2010). 20876032 10.1109/TNSRE.2010.2077654 PMC3037732

[20] Doud, A. J., Lucas, J. P., Pisansky, M. T. & He, B. Continuous Three-Dimensional Control of a Virtual Helicopter Using a Motor Imagery Based Brain-Computer Interface. *PLOS ONE* **6**, e26322 (2011). 22046274 10.1371/journal.pone.0026322 PMC3202533

[21] LaFleur, K. *et al*. Quadcopter control in three-dimensional space using a noninvasive motor imagery based brain-computer interface. *J Neural Eng* **10**, 10.1088/1741-2560/10/4/046003 (2013). 10.1088/1741-2560/10/4/046003 PMC3839680 23735712

[22] Kauhanen, L. *et al*. EEG-Based Brain-Computer Interface for Tetraplegics. *Comput Intell Neurosci* **2007**, 23864 (2007). 18288247 10.1155/2007/23864 PMC2233767

[23] Perdikis, S., Tonin, L., Saeedi, S., Schneider, C. & Millán, J. D. R. The Cybathlon BCI race: Successful longitudinal mutual learning with two tetraplegic users. *PLoS Biol* **16**, e2003787 (2018). 29746465 10.1371/journal.pbio.2003787 PMC5944920

[24] Benaroch, C. *et al*. Long-Term BCI Training of a Tetraplegic User: Adaptive Riemannian Classifiers and User Training. *Front. Hum. Neurosci*. **15** (2021). 10.3389/fnhum.2021.635653 PMC8012558 33815081

[25] Onose, G. *et al*. On the feasibility of using motor imagery EEG-based brain–computer interface in chronic tetraplegics for assistive robotic arm control: a clinical test and long-term post-trial follow-up. *Spinal Cord* **50**, 599–608 (2012). 22410845 10.1038/sc.2012.14

[26] Cervera, M. A. *et al*. Brain‐computer interfaces for post‐stroke motor rehabilitation: a meta‐analysis. *Ann Clin Transl Neurol* **5**, 651–663 (2018). 29761128 10.1002/acn3.544 PMC5945970

[27] Choy, C. S., Cloherty, S. L., Pirogova, E. & Fang, Q. Virtual Reality Assisted Motor Imagery for Early Post-Stroke Recovery: A Review. *IEEE Reviews in Biomedical Engineering* **16**, 487–498 (2023). 35380970 10.1109/RBME.2022.3165062

[28] Kaiser, V. *et al*. Relationship Between Electrical Brain Responses to Motor Imagery and Motor Impairment in Stroke. *Stroke* **43**, 2735–2740 (2012). 22895995 10.1161/STROKEAHA.112.665489

[29] Leamy, D. J. *et al*. An exploration of EEG features during recovery following stroke – implications for BCI-mediated neurorehabilitation therapy. *Journal of NeuroEngineering and Rehabilitation* **11**, 9 (2014). 24468185 10.1186/1743-0003-11-9 PMC3996183

[30] Foong, R. *et al*. Assessment of the Efficacy of EEG-Based MI-BCI With Visual Feedback and EEG Correlates of Mental Fatigue for Upper-Limb Stroke Rehabilitation. *IEEE Transactions on Biomedical Engineering* **67**, 786–795 (2020). 31180829 10.1109/TBME.2019.2921198

[31] Johnson, N. N. *et al*. Combined rTMS and Virtual Reality Brain-Computer Interface Training for Motor Recovery after Stroke. *J Neural Eng* **15**, 016009 (2018). 28914232 10.1088/1741-2552/aa8ce3 PMC5821060

[32] Burle, B. *et al*. Spatial and temporal resolutions of EEG: Is it really black and white? A scalp current density view. *Int J Psychophysiol* **97**, 210–220 (2015). 25979156 10.1016/j.ijpsycho.2015.05.004 PMC4548479

[33] He, B., Sohrabpour, A., Brown, E. & Liu, Z. Electrophysiological Source Imaging: a Noninvasive Window to Brain Dynamics. *Annu Rev Biomed Eng* **20**, 171–196 (2018). 29494213 10.1146/annurev-bioeng-062117-120853 PMC7941524

[34] Zhu, H., Forenzo, D. & He, B. On the Deep Learning Models for EEG-Based Brain-Computer Interface Using Motor Imagery. *IEEE Trans Neural Syst Rehabil Eng* **30**, 2283–2291 (2022). 35951573 10.1109/TNSRE.2022.3198041 PMC9420068

[35] Craik, A., He, Y. & Contreras-Vidal, J. L. Deep learning for electroencephalogram (EEG) classification tasks: a review. *J. Neural Eng.* **16**, 031001 (2019). 30808014 10.1088/1741-2552/ab0ab5

[36] Wu, D., Xu, Y. & Lu, B.-L. Transfer Learning for EEG-Based Brain–Computer Interfaces: A Review of Progress Made Since 2016. *IEEE Transactions on Cognitive and Developmental Systems* **14**, 4–19 (2022).

[37] Schirrmeister, R. T. *et al*. Deep learning with convolutional neural networks for EEG decoding and visualization. *Human Brain Mapping* **38**, 5391–5420 (2017). 28782865 10.1002/hbm.23730 PMC5655781

[38] Lawhern, V. J. *et al*. EEGNet: a compact convolutional neural network for EEG-based brain–computer interfaces. *J. Neural Eng.* **15**, 056013 (2018). 29932424 10.1088/1741-2552/aace8c

[39] Stieger, J. R., Engel, S. A., Suma, D. & He, B. Benefits of deep learning classification of continuous noninvasive brain–computer interface control. *J Neural Eng* **18**, 10.1088/1741-2552/ac0584 (2021). 10.1088/1741-2552/ac0584 PMC9305984 34038873

[40] Song, Y., Zheng, Q., Liu, B. & Gao, X. EEG Conformer: Convolutional Transformer for EEG Decoding and Visualization. *IEEE Trans Neural Syst Rehabil Eng* **PP** (2022). 10.1109/TNSRE.2022.3230250 37015413

[41] Stieger, J. R., Engel, S. A. & He, B. Continuous sensorimotor rhythm based brain computer interface learning in a large population. *Sci Data* **8**, 98 (2021). 33795705 10.1038/s41597-021-00883-1 PMC8016873

[42] Tangermann, M. *et al*. Review of the BCI Competition IV. *Front Neurosci* **6**, 55 (2012). 22811657 10.3389/fnins.2012.00055 PMC3396284

[43] Hossain, K. M., Islam, M. A., Hossain, S., Nijholt, A. & Ahad, M. A. R. Status of deep learning for EEG-based brain–computer interface applications. *Front. Comput. Neurosci*. **16** (2023). 10.3389/fncom.2022.1006763 PMC9885375 36726556

[44] Deng, J. *et al*. ImageNet: A large-scale hierarchical image database. in *2009 IEEE Conference on Computer Vision and Pattern Recognition* 248–255 10.1109/CVPR.2009.5206848 (2009).

[45] Forenzo, D., Zhu, H., Shanahan, J., Lim, J. & He, B. Continuous tracking using deep learning-based decoding for noninvasive brain–computer interface. *PNAS Nexus* **3**, pgae145 (2024). 38689706 10.1093/pnasnexus/pgae145 PMC11060102

[46] Schalk, G., McFarland, D. J., Hinterberger, T., Birbaumer, N. & Wolpaw, J. R. BCI2000: A General-Purpose Brain-Computer Interface (BCI) System. *IEEE Trans. Biomed. Eng.* **51**, 1034–1043 (2004). 15188875 10.1109/TBME.2004.827072

[47] Forenzo, D. & He, B. EEG-BCI Dataset for ‘Continuous Tracking using Deep Learning-based Decoding for Non-invasive Brain-Computer Interface’. *figshare*10.1184/R1/25360300 (2024). 10.1093/pnasnexus/pgae145 PMC11060102 38689706

[48] Cui, W. *et al*. Neuro-GPT: Towards A Foundation Model for EEG. in (2024 IEEE International Symposium on Biomedical Imaging (ISBI), 10.48550/arXiv.2311.03764 (2024).

